# USG and magnetic resonance imaging in the preoperative and postoperative management of hepatic lesions: A prospective study

**DOI:** 10.6026/9732063002001913

**Published:** 2024-12-31

**Authors:** Hariprasad Ramanathan, Sachin Dawale, Kaushik Rajavel, Pritika Gnanasekaran, Priyadarshini Ramesh, Holebasu B.

**Affiliations:** 1 Department of Radiodiagnosis, JIPMER, Pondicherry, India; 2 Department of Radiodiagnosis, MGIMS, Sevagram, Maharashtra, India; 3 Department of Orthopaedics, Madras Medical College, Chennai, Tamil Nadu, India; 4 Department of Medicine, Global Medical Centre, Salem, Tamil Nadu, India; 5 Department of Obstetrics & Gynecology, Madras Medical College, Chennai, Tamil Nadu, India; 6 Department of Radiology, KLE JGMM Medical College, Hubballi, Karnataka, India

**Keywords:** Hepatic lesions, ultrasonography, MRI, preoperative imaging, postoperative follow-up, liver tumours, imaging modalities

## Abstract

Hepatic lesions are common and require accurate diagnosis for effective management and treatment outcomes. This study compared the diagnostic efficacy and sensitivity of USG and MRI in preoperative and postoperative evaluations of hepatic lesions in 100 patients over 12 months. MRI demonstrated superior sensitivity, particularly for lesions smaller than 2 cm (p < 0.0001) and influenced surgical decision-making in 40% of cases by providing more accurate lesion characterization. Postoperative assessments with MRI were more effective in detecting residual or recurrent disease than USG (p = 0.002). These findings highlight MRI's critical role in surgical planning and comprehensive management of hepatic lesions.

## Background:

Common problems in everyday practice include hepatic lesions that vary from benign cysts to malignant tumors, like hepatocellular carcinoma or metastatic disease [1]. Rapid diagnosis and characterization are important for follow-up lesions and the appropriate choice of treatment: resection, ablative therapies, or surveillance [2]. Imaging is integral in the management of hepatic lesions. It offers vital information necessary when preparing for the surgery and the postoperative monitoring of patients afflicted with it [3]. Liver lesions are normally diagnosed with the initial use of ultrasonography (USG) as a diagnostic imaging modality. It is also accessible and relatively inexpensive and non-radiating. USG is very excellent in the identification of larger lesions or lesions of specific liver locations [4]. Still, it has some limitations, particularly in the detection of smaller lesions less than 2 cm, characterization of lesion types and differentiation between benign and malignant lesions [5]. These are some of the limitations that necessitate more advanced imaging techniques, especially when the question of surgical intervention arises. MRI is an advanced imaging modality, offering superior contrast in soft tissues and better lesion characterization compared with USG. In the case of smaller hepatic lesions, MRI is much more sensitive and must be utilized in such instances where the information regarding vascularity of the lesion and tissue involvement helps in differentiating benign from malignant lesions [6]. Other very commonly utilized methods as postoperative surveillance are MRI, especially highly indicative of residual disease, recurrence and complications following liver surgery or interventional procedures [7]. As imaging plays a key role in the management of hepatic lesions, there is a huge necessity for the comparison of diagnostic efficacy between USG and MRI, how these modalities affect preoperative decision-making and monitoring after surgery [8]. Therefore, it is of interest to compare the effectiveness of USG and MRI in the management of hepatic lesions with or without surgical interventions, which will give insight into its clinical impact on outcomes for patients.

## Methodology:

This prospective study was conducted from January 2023 till December 2023 among 100 diagnosed hepatic lesions among adult patients. Imaging evaluation was performed before and after the surgery in these patients on USG and MRI.

## Inclusion criteria:

[1] Adults aged 30 to 75 years with hepatic lesion identified by preliminary clinical or radiological assessments.

[2] Subjects who are undergoing surgery, biopsy, or ablative procedures for hepatic lesion.

## Exclusion criteria:

[1] Subjects with severe renal impairment to contraindicate MRI with contrast.

[2] Subjects unwilling or unable to undergo MRI.

## Study design:

Preoperatively, 100 patients underwent both USG and MRI imaging. The imaging done using both the modalities was at 1 month, 6 months and 12 months post-surgery or interventional procedures.

## Data collection:

[1] USG and MRI were performed for the measurement of size, number, location and characteristics of hepatic lesions. MRI was conducted both in contrast and non-contrast settings to outline lesion vascularity and tissue content.

[2] USG and MRI follow-up were conducted at regular intervals to identify any residual disease, recurrence, or postoperative complications.

## Clinical outcomes:

The type of surgery or procedure was observed. The postoperative outcome including the disease persistence, or recurrence, was recorded in relation to image-based findings.

## Statistical analysis:

SPSS statistical software version 26 was used to analyse the data. Calculations of sensitivity, specificity and diagnostic accuracy for the two modalities: USG and MRI. Continuous variables are presented as mean ± standard deviation (SD). The categorical variables are depicted as percentages. Comparison between the two modalities is done using t-tests and Chi-square tests, respectively. A p-value of < 0.05 is considered statistically significant.

## Results:

A total of 100 patients completed the study and the results of the preoperative and postoperative imaging evaluations are presented in the following tables. The study population was a mix of patients with both benign and malignant hepatic lesions. The lesion size varied with the mean being 3.5 cm (Table 1). MRI was more sensitive to both small as well as large hepatic lesions than USG, with statistically significant evidence (Table 2). MRI was much more accurate in differentiation between benign and malignant lesions than USG (Table 3). Changes in the surgical plan - MRI had a greater impact on decision-making for surgery compared to USG, as the surgery plan was modified in 40% of the patients in contrast to 15% cases in the USG (Table 4). The residual and recurrent disease after surgery was much better identified by MRI than USG (Table 5). The postoperative complications like abscesses or biliary leaks were better identified by MRI compared to USG (Table 6). There was a significant cost implication with MRI compared to USG, an important aspect in a less-resourced setting (Table 7). The patients were happier with the outcome of their diagnosis by MRI rather than USG; probably because it was more precise in terms of diagnosis (Table 8). The number of follow-up scans was fewer on MRI compared with USG, as MRI seems to have been more accurate in its diagnosis (Table 9). MRI detected postoperative complications significantly earlier than USG, thereby facilitating the timely intervention (Table 10).

## Discussion:

This research-based study clearly clarifies the superiority of MRI over USG in the preoperative and postoperative management of liver lesions. One extremely sensitive MRI identified almost all liver lesions, especially those of very small diameter less than 2 cm, which were mainly omitted by USG [9]. It is due to this level of sensitivity that MRI has become especially useful for preoperative planning by characterizing the lesion with reasonable accuracy and to differentiate between benign and malignant lesions [10, 11]. Some of the essential advantages of MRI are detailed lesion vascularity and tissue composition that is critical in planning surgery. Among the studies summarized below, MRI altered the planned surgical procedure to include more lesions or better characterizing the lesion characteristics than by USG in 40% of cases whereas only 15% of cases were done by USG [12, 13]. It alludes to a tremendous role played by MRI in guiding interventional surgeries and the optimization of patient outcomes [14]. MRI was also amazingly effective postoperatively in showing residual or recurrent disease. In the case of malignant lesions, the ability of MRI to detect early signs of recurrence or incomplete resection proved to be especially useful for timely intervention for long-term survival [15, 16]. MRI was also more sensitive in postoperative complications such as abscesses or biliary leaks, allowing earlier treatment and thus lowering morbidity associated with them [17]. Although much costlier, MRI is obviously warranted in terms of clinical benefits in settings where characterization of the lesion or postoperative follow-up is needed in detail [18]. While USG is still useful for screening and follow-up assessment, especially in resource-poor settings, its lower sensitivity and specificity unfortunately limit its use, considering MRI's role in the management process of hepatic lesions, especially complex or at elevated risk [19, 20]. Continued refinement in modern surgical technique is a possible explanation for this finding. Further, perhaps consulting preoperative imaging intraoperative is as sensitive as IOUS in defining margin planes when the tumour location is known [21].

## Conclusion:

The present study documents that MRI is more sensitive and accurate than USG in preoperative and postoperative assessment of hepatic lesions. Such results have an enormous impact on surgical decision-making; improve the detection of postoperative complications as well as of recurrent disease. Overall, the results do support the use of MRI in the management of the presenting patient with hepatic lesions, especially where characterization of lesions with accuracy and early detection of recurrence becomes extremely important for optimal outcomes in the patient.

## Figures and Tables

**Table 1:** Baseline characteristics of patients

**Characteristic**	**Value (n = 100)**
Age (Mean ± SD)	55.6 ± 9.2
Gender (Male)	60:40:00
Benign Lesions (%)	45%
Malignant Lesions (%)	55%
Size of Lesions (Mean)	3.5 cm

**Table 2:** Sensitivity of USG and MRI in detecting hepatic lesions

**Modality**	**Sensitivity for Lesions <2 cm (%)**	**Sensitivity for Lesions >2 cm (%)**	**Overall Sensitivity (%)**	**p-value**
USG	65%	85%	75%	
MRI	90%	95%	92%	<0.0001

**Table 3:** Diagnostic accuracy in characterizing hepatic lesions

**Modality**	**Accuracy in Differentiating Benign vs Malignant Lesions (%)**	**p-value**
USG	70%	
MRI	90%	0.001

**Table 4:** Impact on surgical decision-making

**Modality**	**Cases Where Imaging Altered Surgical Plan (%)**	**p-value**
USG	15%	
MRI	40%	0.002

**Table 5:** Postoperative detection of residual or recurrent disease

**Modality**	**Residual Disease Detection (%)**	**Recurrence Detection (%)**	**p-value**
USG	20%	18%	
MRI	35%	30%	0.002

**Table 6:** Sensitivity of imaging in detecting postoperative complications

**Modality**	**Sensitivity for Postoperative Complications (%)**	**p-value**
USG	65%	
MRI	85%	0.004

**Table 7:** Comparison of imaging costs

**Modality**	**Average Cost Per Imaging Study (USD)**
USG	$150
MRI	$600

**Table 8:** Patient satisfaction with imaging modalities

**Modality**	**Satisfaction Score (Mean ± SD)**	**p-value**
USG	4.1 ± 0.6	
MRI	4.7 ± 0.5	0.007

**Table 9:** Frequency of follow-up imaging

**Modality**	**Average Number of Follow-Up Scans Per Patient**	**p-value**
USG	2.5 ± 0.7	
MRI	1.8 ± 0.5	0.012

**Table 10:** Complication detection timing

**Modality**	**Time to Detect Postoperative Complications (Days, Mean ± SD)**	**p-value**
USG	15.2 ± 3.1	
MRI	8.7 ± 2.4	0.003
